# Respiratory motion variability of primary tumors and lymph nodes during radiotherapy of locally advanced non-small-cell lung cancers

**DOI:** 10.1186/s13014-015-0435-3

**Published:** 2015-06-14

**Authors:** Nuzhat Jan, Geoffrey D. Hugo, Nitai Mukhopadhyay, Elisabeth Weiss

**Affiliations:** Department of Radiation Oncology, Virginia Commonwealth University, 401 College Street, PO Box 980058, Richmond, VA 23298 USA; Department of Biostatistics, Virginia Commonwealth University, One Capital Square, Richmond, VA 23298 USA

**Keywords:** Non-small-cell lung cancer, Respiratory motion, Primary tumor, Lymph nodes

## Abstract

**Background and purpose:**

The need for target adjustment due to respiratory motion variation and the value of carina as a motion surrogate is evaluated for locally advanced non-small-cell lung cancer.

**Material and methods:**

Using weekly 4D CTs (with audio-visual biofeedback) of 12 patients, respiratory motion variation of primary tumors (PT), lymph nodes (LN) and carina (C) were determined.

**Results:**

Mean (SD) 3D respiratory motion ranges of PT, LN and C were 4 (3), 5 (3) and 5 (3) mm. PT and LN (*p* = 0.003), and LN and C motion range were correlated (*p* = 0.03). Only 20 %/5 % of all scans had variations >3 mm/5 mm. Large respiratory motion range on the initial scan was associated with larger during-treatment variations for PT (*p* = 0.03) and LN (*p* = 0.001).

Mean (SD) 3D relative displacements of PT-C, LN-C and PT-LN were each 6 (2) mm. Variations of displacements >3 mm/5 mm were observed in 28 %/6 % of scans for PT-LN, 20 %/9 % for PT-C, and 20 %/8 % for LN-C.

**Conclusions:**

Motion reassessment is recommended in patients with large initial motion range. Relative motion-related displacements between PT and LN were larger than PT and LN motion alone. Both PT and C appear to be comparable surrogates for LN respiratory motion.

## Background

The respiratory motion of primary lung tumors (PT) and involved lymph nodes (LN) has been studied extensively showing motion ranges up to 3.5 cm for PT [1–6] and 1.5 cm for LN [7–13]. Respiratory motion of locally advanced non-small-cell lung cancer (LA-NSCLC) is commonly assessed prior to therapy, assuming stable respiratory conditions throughout the course of treatment. Several longitudinal investigations of PT motion [3, 14–18] reported both stable motion as well as in- or decrease. These analyses extended over varying time spans and were usually limited to 2 or 3 repeat scans during treatment. Temporal variations of mediastinal LN motion have rarely been investigated [13, 19] and covered only short time periods, except for one study using implanted markers as surrogates for mediastinal LN and daily 4D CBCT imaging [11].

Target volumes for LA-NSCLC need to ensure coverage of both PT and involved LN over the whole radiotherapy course. The primary goal of this longitudinal study is therefore to investigate PT and LN respiratory motion variation together over the period of a conventional radiation treatment. Investigations so far have focused on either PT or LN and did not analyze the geometric relationship during respiration between both parts of the complex target in LA-NSCLC. Knowledge of PT, LN and PT relative to LN (PT-LN) motion is also relevant for gated therapies to select appropriate phases with ideally little respiratory displacement. In addition, information on PT, LN and PT-LN motion variation is a prerequisite for the development of target tracking in LA-NSCLC which is at present only used for early stage lung cancer without LN involvement. This study also investigates carina (C) as a surrogate for LN respiratory motion, as LNs are often not readily visible on onboard imaging.

## Materials and methods

### Patients and imaging

Twelve consecutively enrolled patients with stage IIIA locally advanced non-small-cell lung cancer underwent weekly 4D CT imaging (4–8 weekly scans per patient) on a prospective IRB-approved imaging protocol (Virginia Commonwealth University IRB). Primary tumors were located in the middle and lower lobes in 7 patients, analyzed LNs were in region 4 in 8, and regions 1, 2 and 7 in the remainder of patients [20]. The average volumes on the planning scan were 77 cm^3^ (range 7–392 cm^3^) for PT and 5 cm^3^ (range 1–15 cm^3^) for LN. The treatment was concurrent radiochemotherapy to a total dose of 64.8-70 Gy using daily 1.8 or 2 Gy fractions. A total of 65 4D CTs, each divided into 10 phase bins using phase-based sorting (Brilliance Big Bore, Philips Medical Systems, Andover, MA), were acquired with a slice thickness of 3 mm, 512 × 512 axial resolution and a 50–60 cm field of view. Audiovisual biofeedback was used throughout imaging and treatment [21, 22].

### Contouring

Primary tumor (PT), involved lymph nodes (LN) and carina (C) were manually contoured by one physician on the 10 respiratory phases (0, 10, 20 ….., 90 % with 0 % being end- inspiration and 50-70 % being end-expiration phase) of each 4D CT using a commercial treatment planning system (Pinnacle 8.1, Phillips, Fitchburg, WI). No contrast was used for these scans. All LN and the parts of PT neighboring mediastinum, diaphragm and chest wall were contoured in the default mediastinal window, primary tumors surrounded by lung tissue were delineated in the lung window. The largest lymph node in each patient was used for data analysis. Large emphasis was given to high quality contouring given the expected small inter-phase positional variations. Manual contouring was aided through copying and editing contours between phases and through the use of individualized contouring templates to reduce contour variability between phases and weekly scans. Peer review was performed for all contours.

### Data analysis

#### Absolute respiratory motion range and relative respiration-related displacements

The center of mass (centroid) positions of PTs, LNs and Cs were recorded for all phases on all scans (total of 650 3D CT scans). The range of respiratory motion of all three structures was determined by calculating the largest differences of the centroid positions on the 10 phase bins of each 4D CT for the three cardinal directions, x (lateral), y (anteroposterior) and z (superior-inferior) relative to the centroid position in phase 0 %. The largest three dimensional (3D) displacement vector magnitude was determined from the square root of the sum of the squared x-, y- and z-displacements for each phase bin per 4D CT. From the maximum ranges of motion on repeated 4D CTs, the individual patient means, and from averaging the patient means over all patients, the population means were determined. The largest displacements in the three cardinal directions might occur in different breathing phases. The resulting 3D vector based on these maximum displacements would be physiologically unrealistic, as there actually is no breathing phase that showed such a high 3D displacement. Therefore, after calculation of the 3D displacement vector for each phase bin of each scan, the largest 3D vector per scan was selected from the 10 phase bins and was typically smaller than expected from the maximum displacements in the individual directions. The patient means of the 3D displacement vectors were determined by selecting the largest 3D vector per scan and averaging over all scans per patient. Phase bins with the largest 3D vector varied between scans. Patient means were averaged to obtain the population means of 3D displacement vectors.

Using the centroid coordinates of each structure, relative respiration-related displacements of PT-LN, PT-C, and LN-C were calculated for each phase of all 4D CTs for all three cardinal directions by subtracting the x-, y- and z-positions of the two respective centroids for each phase and determining the largest difference per scan. Mean patient-specific relative displacements were averaged over all patients to obtain the population means for all directions and 3D vectors. Similar to absolute displacements, the 3D vectors of relative displacements were selected from the phase bin with the largest 3D displacement vector per scan.

#### Time trends in respiratory motion range

Longitudinal variations in respiratory motion ranges during a course of radiotherapy were calculated as differences in the average ranges of motion between the initial planning scan (week 1) and subsequent scans. To determine the spectrum of variability within the population, scans with >1/3/5 mm variation in the respiratory range and relative displacements of PT, LN and C compared to the planning scan were identified.

### Statistics

Longitudinal data were modeled using a linear mixed effect model, thereby allowing for a random effect of individual patient data on the population model. Fixed effects in the model were absolute and relative motion per week and week 1 displacement. Correlation among repeated measures of the same patients was modeled using a compound symmetric covariance matrix based on optimal Akaike information criteria (AIC) and successful convergence of the optimization process [23]. All analyses were done using PROC MIXED in SAS v9.3. Results were assumed to be statistically significant for p < 0.05 for two-sided tests. Power calculations were performed for all analyses and showed at least 80 % power for all significant results.

## Results

### Absolute and relative respiratory motion range

For average range of motion for PT, LN and C see Table 1. While the range of PT motion was not significantly associated with C motion (*p* = 0.08), it was significantly associated with LN motion (*p* = 0.003). Also, LN and C motion were positively correlated (*p* = 0.03). Individual 3D patient means of respiratory motion range (all scans over the treatment course per patient) for PT, LN and C ranged from 0 to 7, 1 to 9, and 1 to 11 mm, respectively, indicating large interpatient variations in respiratory motion.

**Table 1:** Average range of respiratory motion for all scans and patients

Average respiratory motion (± standard deviation) in mm				
	x	y	z	3D
PT	2 (1)	2 (2)	4 (3)	4 (3)
LN	2 (2)	2 (2)	4 (3)	5 (3)
C	2 (2)	2 (2)	4 (3)	5 (3)
PT-LN	3 (3)	3 (2)	3 (3)	6 (2)
PT-C	2 (2)	3 (2)	5 (3)	6 (2)
LN-C	3 (3)	3 (2)	4 (2)	6 (2)

C: Carina; LN: Lymph nodes; PT: Primary tumor

For average range of relative respiration-related displacements of PT-LN, PT-C and LN-C see Table 1. Individual 3D patient means for these relative position changes ranged from 2 to 10, 2 to 12, and 4 to 10 mm, respectively.

### Time trends in respiratory motion range

**Fig. 1:**
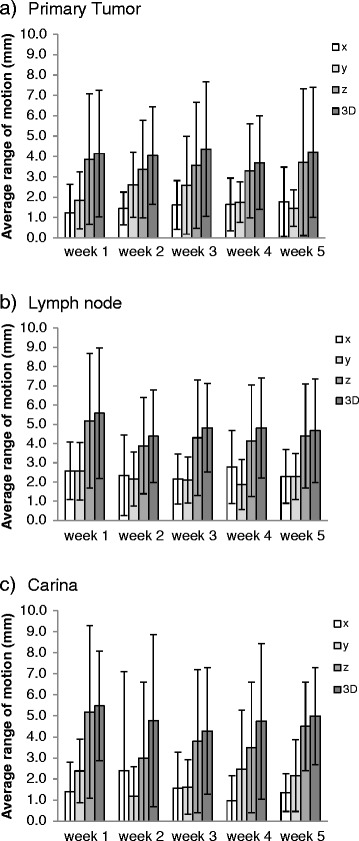
Variation of the range of motion during radiotherapy. Means and standard deviations of the study population in the three cardinal directions and as 3D vectors per week for **a** Primary Tumor, **b** Lymph Node and **c** Carina

**Fig. 2:**
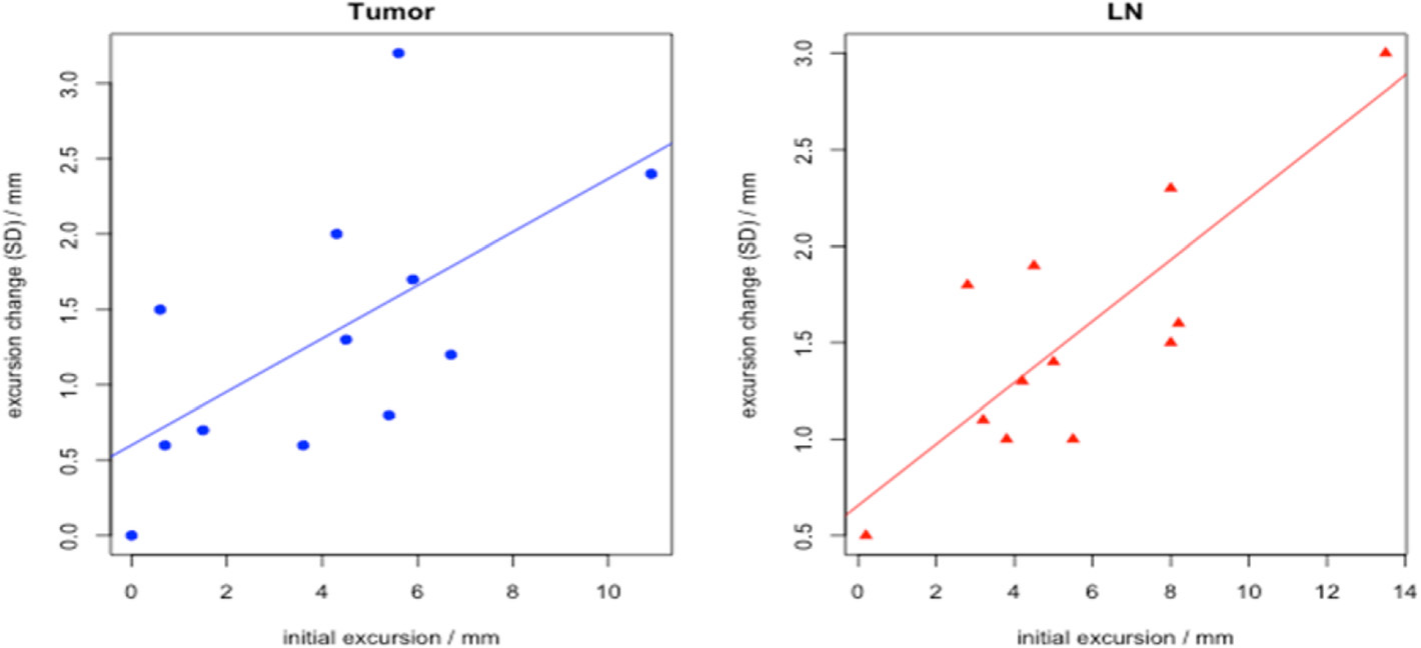
Relation between week 1 range of motion and longitudinal variability. Larger range of motion of primary tumor (*p* = 0.03) and lymph node (*p* = 0.001) on the week 1 scan is associated with more motion variability on subsequent scans. For each patient, the initial range of motion (x-axis) versus the standard deviation of the motion range on subsequent scans (y-axis) is shown

**Fig. 3:**
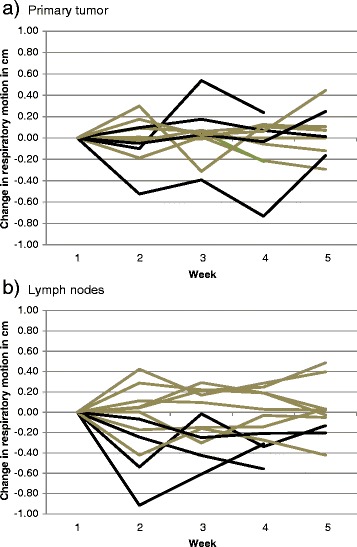
Variation of respiration-related 3D displacements relative to week 1 (planning scan). Black lines represent patients with either ≥6 mm primary tumor motion range in **a** or ≥8 mm lymph node motion range in **b** in week 1. Patients with larger motion range on the initial week1 scan were more likely to have a > 5 mm change in motion range. Changes in respiratory motion in weeks 2–5 are normalized to the week 1motion range

While the mean difference between week 1 and week 5 range of respiratory motion was less than 1 mm for PT, LN and C for all directions, large interpatient variability was observed (Fig. 1). 3D variations >1 mm were observed in all but one patient. A change of motion amplitude >3 mm relative to the planning scan was identified in 11 % of scans for PT and 20 % for LN and C, of which 3, 5 and 9 % were increases. Variations >5 mm were rare with 5 % for PT and LN, and 11 % for C, of which up to 5 % were increases (Table 2). Larger respiratory motion range on the initial scan was associated with more variation in subsequent weeks for PT (*p* = 0.03), LN (*p* = 0.001), but not C (*p* = 0.3) (Fig. 2). Two of four patients with ≥6 mm PT motion, and 3 of 4 patients with ≥8 mm LN motion amplitude on the initial scan had >5 mm variations of respiratory amplitude compared to none below these motion ranges. As shown in Fig. 3, no clear time trends towards enlarging or diminishing motion ranges were observed for PT, whereas LN motion ranges appeared to decrease. These observations need to be confirmed in larger patient cohorts.

**Table 2:** Variability of respiratory motion during the radiotherapy course for all patients (total 12) and scans (total 65)

> 3 mm change/increase in respiratory motion range and relative displacement								
	x		y		z		3D	
	Patients	Scans	Patients	Scans	Patients	Scans	Patients	Scans
PT	1/1	1/1	1/1	1/1	3/1	5/1	4/2	7/2
LN	1/0	1/0	1/1	1/1	6/2	9/4	6/2	13/3
C	3/2	8/5	4/1	8/1	4/2	6/2	7/3	13/6
PT-LN	3/3	3/3	4/4	4/4	8/2	15/2	9/8	18/10
PT-C	3/3	7/4	3/2	7/3	7/4	13/5	7/3	13/4
LN-C	4/3	11/7	3/2	3/2	5/3	6/4	7/5	13/9
>5 mm change/increase in respiratory motion range and relative displacement								
	x		y		z		3D	
	Patients	Scans	Patients	Scans	Patients	Scans	Patients	Scans
PT	1/1	1/1	0/0	0/0	1/1	2/2	2/1	3/1
LN	0/0	0/0	1/1	1/1	2/0	3/0	3/0	3/0
C	0/0	0/0	1/1	1/1	1/1	3/2	2/1	7/3
PT-LN	3/3	3/3	2/2	2/2	3/1	4/1	3/3	4/3
PT-C	1/1	1/1	1/1	1/1	3/1	5/1	3/1	6/1
LN-C	3/2	5/3	1/1	2/2	3/2	3/2	3/2	5/2

C: Carina; LN: Lymph nodes; PT: Primary tumor

**Fig. 4:**
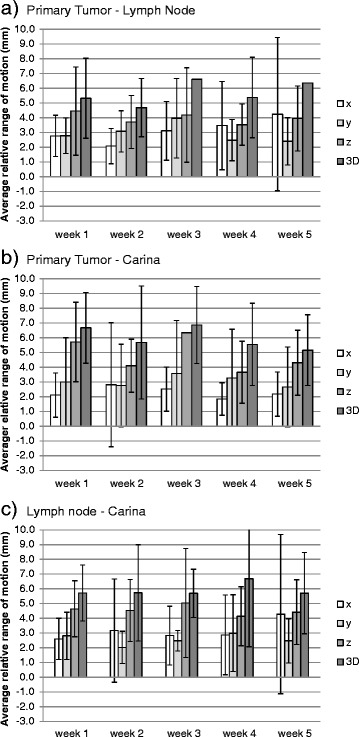
Variation of the relative respiration-related displacements. Means and standard deviations of the study population in the three cardinal directions and as 3D vectors per week for **a** Primary Tumor - Lymph Node, **b** Primary Tumor - Carina, **c** Lymph Node - Carina

**Fig. 5:**
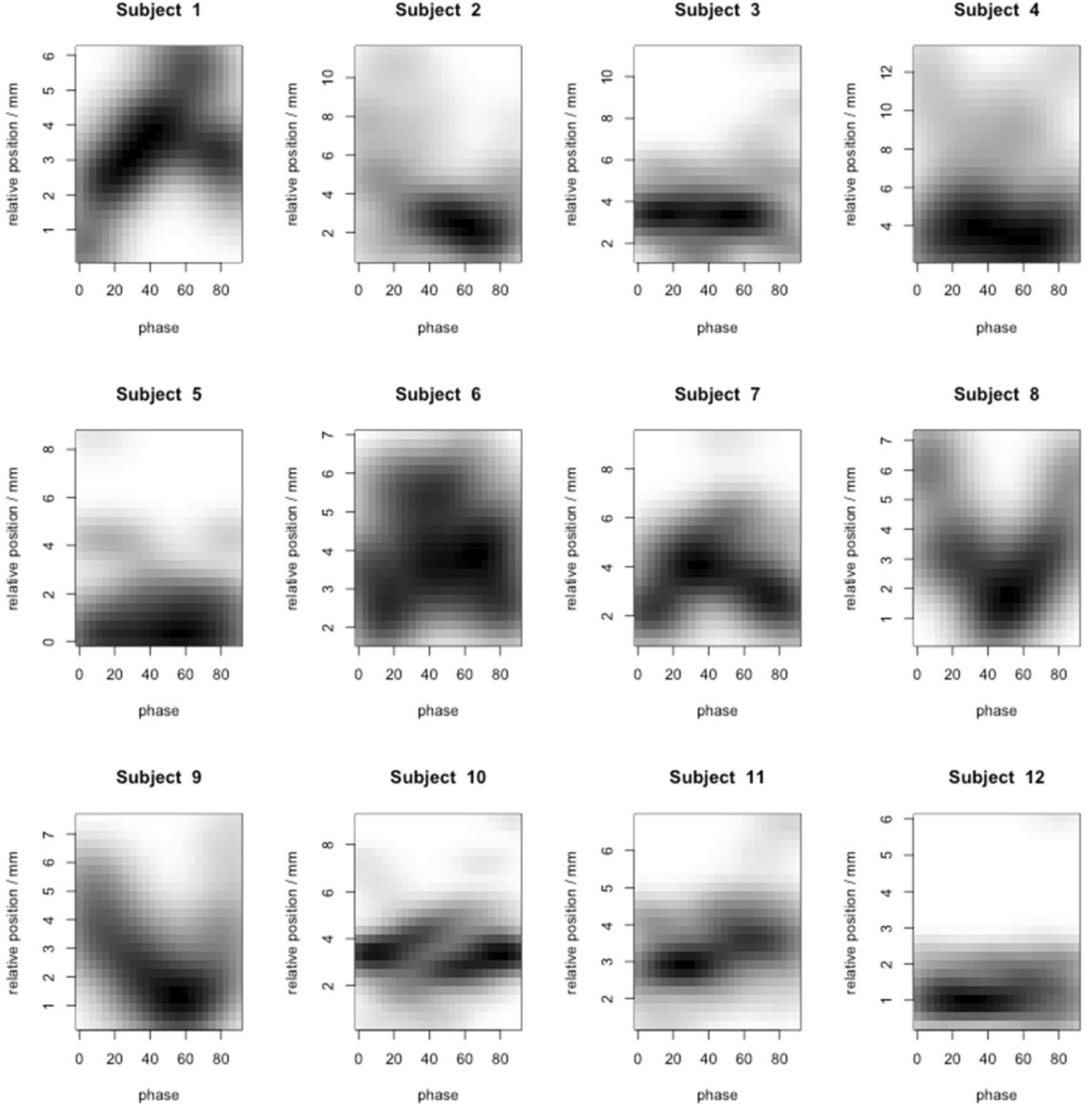
Occupancy maps of relative respiratory motion-related displacements between primary tumors and lymph nodes for individual patients. Relative 3D displacements between primary tumor and lymph node relative to respiration phase 0–90 (0: end inspiration, 50: end expiration) are shown for all scans (4–8) per patient. Values were interpolated between neighboring phases. Darker color means that the respective magnitude of displacement is observed more frequently during respiration. While there is only little displacement with phase, e.g., in patients 3 and 12, other patients show larger variations of displacement during respirations which are stable on repeat scans, e.g., patients 7 and 8. Most patients show variable amounts of displacement between scans

The mean difference between week 1 and week 5 relative motion-related displacements was ≤ 1 mm, except for PT-C where the relative motion was smaller by 1.5 mm in week 5 (Fig. 4). Over the course of therapy, variations in the relative motion-related displacements >3 mm relative to the planning CT were identified in 28 % of scans for PT-LN, 20 % for PT-C, and 20 % for LN-C. Variations >5 mm were seen in 6 % for PT-LN, 9 % for PT-C, and 8 % for LN-C (Table 2). Larger relative displacement on the initial scan was associated with more variation in subsequent weeks, but was not statistically significant. For PT-LN, the 3 patients with the largest displacements on the initial scans had >3 mm displacements in 70 % of the subsequent scans. For PT-C, of 3 patients with >5 mm variations on subsequent scans, 2 patients had >10 mm displacement on the initial scan. Figure 5 displays occupancy maps of PT-LN displacement of all scans per patient. Most patients show variable amounts of displacement over time indicating a potential benefit of reassessment of respiratory motion behavior.

## Discussion

### Average motion range

This study provides new information on correlated PT and associated LN respiratory motion during radiotherapy. The magnitude of motion averaged over the treatment course was comparable for PT and LN with large interpatient variations. As observed in other reports, the major trajectory of motion was in craniocaudal direction both for PT and LN [4, 7, 9, 12, 13, 15, 17, 19]. The amount of respiratory motion with on average 4 mm in z-direction for PT and LN in our study appeared smaller than in other reports with average motion ranges up to 11 mm for PT and up to 7 mm for LN [8, 9, 12]. Larger PT motion ranges have been reported for small peripheral tumors [4, 5] and lower lobe tumors [3, 15, 24], whereas in our population of stage IIIA lung cancers no extreme motion ranges were observed, likely due to adherence or invasion of the mediastinum. It is well known that the range of LN motion depends on the LN location [9, 13]. In the present analysis, LN location was, with one exception, supracarinal. The observed 3D motion of 5 mm agrees well with reports of about 5 mm motion for region 4 LNs [9, 11, 19]. The average motion for carina was the same as for LN and comparable to 5 mm reported by van der Weide [18].

### Relative respiration-related displacements

Relative respiration-related displacement has so far not been investigated except for a report by Piet et al. [10] who identified relative motion between C and LN as a potential cause for low yield rates with transbronchial biopsy. The displacement between LN-C of 5 mm in z-direction was similar to the 4 mm observed in our study. Investigation of relative motion is of particular interest for the development of tumor tracking techniques for LA-NSCLC [25, 26]. Tracking of LA-NSCLC is challenging as it requires simultaneous tracking of both PT and LN, with LN usually difficult to identify on planar x-ray or CBCT images. While LN motion was positively correlated with C motion indicating that C might be a good surrogate for LN respiratory motion, as C is readily visible on standard set up imaging, such as kV x-rays and CBCT, relative respiration-related displacements between LN and C were comparable to LN and PT. Therefore, PT and C appear to be comparable surrogates for respiratory motion of LN. Investigating the motion properties of PT, LN and surrogates relative to each other is of interest for the development of motion models in the complex geometries of lung cancer. In addition, information on relative respiration-related displacements is clinically relevant for the selection of appropriate breathing phases for gating techniques where typically end expiration phases with little motion are selected.

### Temporal variations in motion range

High rates of ITV misses during radiotherapy have been described by Mohammed [27] due to position, motion, shape and volume changes. The present study focuses on motion variations, showing that all but one patient had change in motion range >1 mm. Increased ranges of motion >3 mm relative to the planning scan, however, were rare despite the observed volume changes during treatment. The observed variations of relative displacements were also small. PTV margins of 5 mm covered 98 % of PT, 95 % of C and 100 % of LN and 95 % of PT-LN, 98 % of PT-C and 97 % of LN-C relative motion variations assuming use of an ITV to cover initial motion ranges and free breathing situations. In situations with larger motion range on the planning scan, margins might need to be adjusted to cover variations of respiratory motion during therapy. Variations of absolute and relative motion >5 mm were significantly more frequent in patients with larger initial motion range suggesting that patients with large initial motion range might benefit more from reevaluation. So far, few studies have performed repeated motion analysis of PTs over the treatment course. Britton et al. [15] found increased PT mobility on weekly 4D CTs and suggested repeated 4D CT for reassessment of ITVs. Michalski et al. [3] repeated 4D CTs after an average of 34 days and observed reproducible target motion in 87 %. Redmond et al. [17] analyzed PT motion on 2 repeat 4D CTs at 30 and 50 Gy and found no significant motion variation. Clearly, differences in the time periods of reassessment, use or avoidance of biofeedback strategies and the overall observation period have resulted in these seemingly contradictory findings.

Time trends in lymph node motion have rarely been analyzed. While Thomas et al. [13] evaluated whole LN regions, Bosmans et al. [19] analyzed individual LN motion over the first 2 weeks of treatment and found only minor decrease in average motion from 5.6 mm to 5.3 mm. Using implanted markers and daily imaging, Schaake et al. [11] also found minimal average motion changes of <1 mm which is in agreement with our findings. While population-based analyses might reveal small variations, for consideration of re-planning and adaptation, individual patient variations are important. As demonstrated in this study, patients with large initial motion have also larger variations during treatment and therefore might benefit from reassessment of their ITVs. Given the week-to-week variations in motion range, no optimum time point for reassessment can be defined. The need for reassessment is influenced by the scenario selected for differential (PT and LN or C) motion management in LA-NSCLC, which depends on the combination of image guidance strategy (e.g., repeated x-ray imaging, 4D CBCT), respiration management (e.g., tracking, breath hold, free breathing) and patient-specific factors (e.g., location and number of involved lymph nodes, availability of implanted markers in PT and/or LN).

Assuming a scenario of “real-time” PT tracking for LA-NSCLC during free breathing, day-to-day and intrafraction variations of the respiratory motion range would be accounted for during the tracking process. Only a small margin would be required to cover the time lag between the assessment of the target position and adjustments of the treatment field. To cover the LN motion in this scenario, an ITV based on appropriate volumetric scans, e.g., all breathing phases of a 4D CT planning scan, should be generated. Based on our findings, both increases in LN motion range and relative displacements of LN relative to PT or C > 3 mm are rare and are usually covered by a 5 mm PTV margin with image guidance of either PT or C. As an alternative to tracking the PT, tracking C or even LN (provided they are made visible by implanted markers) would be an option if large ITVs in the mediastinum due to large LN motion are prohibitive with regards to normal tissue toxicity. Ideally, all involved targets, PT and LNs, should be tracked independently for optimum target coverage and normal tissue sparing. In scenarios without tracking that use (4D) CBCTs for motion assessment, ITVs of PT and LNs on the initial scan cover both absolute and relative motion ranges. As shown in our study, absolute and relative motion increases >5 mm were observed for PT in one scan (2 % of all scans) and for PT-LN in 3 scans (5 % of all scans). As absolute motion and relative displacement are related, margins of 5 mm should be sufficient to cover both absolute and relative variations in respiratory motion. This is, however, an estimate which ignores other important sources of uncertainty such as delineation error and the quadratic nature of error summation for margin generation.

In the present study, motion range was measured on 4D CTs that cover only few respiratory cycles, potentially underestimating actual intrafraction motion variations. It has been shown, however, that motion ranges in general remain stable during one fraction [14, 16]. Both 4D CT imaging artifacts and contouring variability might have influenced the present data. Several measures as described above were applied to improve image quality and contouring consistency. Most importantly, only one physician performed all contouring to avoid interobserver variation.

## Conclusions

Despite relevant volume shrinkage, the majority of respiratory motion variations were small. Reassessment of respiratory motion is, however, recommended in patients with large initial motion range. Relative respiration-related displacements were on average larger than PT and LN respiratory motion alone. C and PT appeared to be comparable surrogates for determining LN position. Information on relative displacements is relevant for gated treatments and for the development of tracking in LA-NSCLC and should be investigated further.

## References

[1] Keall PJ, Mageras GS, Balter JM, Emery RS, Forster KM, Jiang SB (2006). The management of respiratory motion in radiation oncology report of AAPM task group 76. Med Phys.

[2] Mageras GS, Pevsner A, Yorke ED, Rosenzweig KE, Ford EC, Hertanto A (2004). Measurement of lung tumor motion using respiration-correlated CT. Int J Radiat Oncol Biol Phys.

[3] Michalski D, Sontag M, Li F, de Andrade RS, Uslene I, Brandner ED (2008). Four-dimensional computed tomography-based interfractional reproducibility study of lung tumor intrafractional motion. Int J Radiat Oncol Biol Phys.

[4] Shirato H, Suzuki K, Sharp GC, Fujita K, Onimaru R, Fujino M (2006). Speed and amplitude of lung tumor motion precisely detected in four-dimensional setup and in real-time tumor-tracking radiotherapy. Int J Radiat Oncol Biol Phys.

[5] Sonke JJ, Lebesque J, van Herk M (2008). Variability of four-dimensional computed tomography patient models. Int J Radiat Oncol Biol Phys.

[6] Weiss E, Wijessoriya K, Dill SV, Keall PJ (2007). Tumor and normal tissue motion in the thorax during respiration: Analysis of volumetric and positional variations using 4D CT. Int J Radiat Oncol Biol Phys.

[7] Donnelly ED, Parikh PJ, Lu W, Zhao T, Lechleiter K, Nystrom M (2007). Assessment of intrafraction mediastinal and hilar lymph node movement and comparison to lung tumor motion using four-dimensional CT. Int J Radiat Oncol Biol Phys.

[8] Jenkins P, Salmon C, Mannion C (2005). Analysis of the movement of calcified lymph nodes during breathing. Int J Radiat Oncol Biol Phys.

[9] Pantarotto JR, Piet AH, Vincent A, van-Sörnsen de-Koste JR, Senan S (2009). Motion analysis of 100 mediastinal lymph nodes: potential pitfalls in treatment planning and adaptive strategies. Int J Radiat Oncol Biol Phys.

[10] Piet AH, Lagerwaard FJ, Kunst PW, Slotman BJ, Senan S (2007). Can mediastinal nodal mobility explain the low yield rates for transbronchial needle aspiration without real-time imaging?. Chest.

[11] Schaake EE, Belderbos JS, Buikhuisen WA, Rossi MM, Burgers JA, Sonke JJ (2012). Mediastinal lymph node position variability in non-small cell lung cancer patients treated with radical irradiation. Radiother Oncol.

[12] Sher DJ, Wolfgang JA, Niemierko A, Choi NC (2007). Quantification of mediastinal and hilar lymph node movement using four-dimensional computed tomography scan: implications for radiation treatment planning. Int J Radiat Oncol Biol Phys.

[13] Thomas JG, Kashani R, Balter JM, Tatro D, Kong FM, Pan CC (2009). Intra and interfraction mediastinal nodal region motion: implications for internal target volume expansions. Med Dosim.

[14] Bissonnette JP, Franks KN, Purdie TG, Moseley DJ, Sonke JJ, Jaffray DA (2009). Quantifying interfraction and intrafraction tumor motion in lung stereotactic body radiotherapy using respiration-correlated cone beam computed tomography. Int J Radiat Oncol Biol Phys.

[15] Britton KR, Starkschall G, Tucker SL, Pan T, Nelson C, Chang JY (2007). Assessment of gross tumor volume regression and motion changes during radiotherapy for non-small-cell lung cancer as measured by four-dimensional computed tomography. Int J Radiat Oncol Biol Phys.

[16] Guckenberger M, Wilbert J, Meyer J, Baier K, Richter A, Flentje M (2007). Is a single respiration-correlated 4D-CT study sufficient for evaluation of breathing motion?. Int J Radiat Oncol Biol Phys.

[17] Redmond KJ, Song DY, Fox JL, Zhou J, Rosenzweig CN, Ford E (2009). Respiratory motion changes of lung tumors over the course of radiation therapy based on respiration-correlated four-dimensional computed tomography scans. Int J Radiat Oncol Biol Phys.

[18] Van der Weide L, van Sörnsen de Koste JR, Lagerwaard FJ, Vincent A, van Triest B, Slotman BJ (2008). Analysis of carina position as surrogate marker for delivering phase-gated radiotherapy. Int J Radiat Oncol Biol Phys.

[19] Bosmans G, Van Baardwijk A, Dekker A, Ollers M, Wanders S, Boersma L (2008). Time trends in nodal volumes and motion during radiotherapy for patients with stage III non-small-cell lung cancer. Int J Radiat Oncol Biol Phys.

[20] Mountain CF, Dresler CM (1997). Regional lymph node classification for lung cancer staging. Chest.

[21] Venkat RB, Sawant A, Suh Y, George R, Keall PJ (2008). Development and preliminary evaluation of a prototype audiovisual biofeedback device incorporating a patient-specific guiding waveform. Phys Med Biol.

[22] Jan N, Balik S, Hugo GD, Mukhopadhyay N, Weiss E (2014). Interfraction displacement of primary tumor and involved lymph nodes relative to anatomical landmarks in image–guided radiotherapy of locally advanced lung cancer. Int J Radiat Oncol Biol Phys.

[23] Fitzmaurice GM, Laird NM, Ware JH (2011). Applied Longitudinal Analysis.

[24] Seppenwoolde Y, Shirato H, Kitamura K, Shimizu S, van Herk M, Lebesque JV (2002). Precise and real-time measurement of 3D tumor motion in lung due to breathing and heartbeat, measured during radiotherapy. Int J Radiat Oncol Biol Phys.

[25] Sawant A, Smith RL, Venkat RB, Santanam L, Cho B, Poulsen P (2009). Toward submillimeter accuracy in the management of intrafraction motion: the integration of real-time internal position monitoring and multileaf collimator target tracking. Int J Radiat Oncol Biol Phys.

[26] Poels K, Depuydt T, Verellen D, Engels B, Collen C, Heinrich S (2013). A complementary dual-modality verification for tumor tracking on a gimbaled linac system. Radiother Oncol.

[27] Mohammed N, Kestin L, Grills I, Shah C, Glide-Hurst C, Yan D (2012). Comparison of IGRT registration strategies for optimal coverage of primary lung tumors and involved lymph nodes based on multiple four-dimensional CT scans obtained throughout the radiotherapy course. Int J Radiat Oncol Biol Phys.

